# A Rab39-Klp98A-Rab35 endocytic recycling pathway is essential for rapid Golgi-dependent furrow ingression

**DOI:** 10.1242/dev.201547

**Published:** 2023-08-17

**Authors:** Hui Miao, Megan Millage, Katherine R. Rollins, J. Todd Blankenship

**Affiliations:** Department of Biological Sciences, University of Denver, Denver, CO 80208, USA

**Keywords:** *Drosophila*, Rab39, Klp98A, Endocytic recycling, Furrow ingression, Syncytium

## Abstract

Ingression of the plasma membrane is an essential part of the cell topology-distorting repertoire and a key element in animal cell cytokinesis. Many embryos have rapid cleavage stages in which they are furrowing powerhouses, quickly forming and disassembling cleavage furrows on timescales of just minutes. Previous work has shown that cytoskeletal proteins and membrane trafficking coordinate to drive furrow ingression, but where these membrane stores are derived from and how they are directed to furrowing processes has been less clear. Here, we identify an extensive Rab35/Rab4>Rab39/Klp98A>trans-Golgi network (TGN) endocytic recycling pathway necessary for fast furrow ingression in the *Drosophila* embryo. Rab39 is present in vesiculotubular compartments at the TGN where it receives endocytically derived cargo through a Rab35/Rab4-dependent pathway. A Kinesin-3 family member, Klp98A, drives the movements and tubulation activities of Rab39, and disruption of this Rab39-Klp98A-Rab35 pathway causes deep furrow ingression defects and genomic instability. These data suggest that an endocytic recycling pathway rapidly remobilizes membrane cargo from the cell surface and directs it to the trans-Golgi network to permit the initiation of new cycles of cleavage furrow formation.

## Teaser

**Summary:** Identification of an endocytic recycling pathway that permits the rapid re-utilization of membrane for the formation of pseudocleavage furrows in the early *Drosophila* embryo.

## INTRODUCTION

The ability of a cell to drive the ingression of select portions of the plasma membrane is a crucial activity in a variety of cell types and tissue contexts. Indeed, plasma membrane ingression is key to the topological changes that underlie the formation of a dividing furrow that drives cytokinesis and daughter cell separation. In animal cells, furrow ingression is directed by a combination of cytoskeletal force generation and membrane trafficking events (reviewed by Pollard, 2010; Neto et al., 2011; Schiel and Prekeris, 2013). Depending on the final dimensions of the daughter cells, there is often a requirement for membrane growth during this division process – in addition, this membrane addition may be polarized to direct essential activities required for cytokinesis and abscission (Knutton et al., 1975; Erickson and Trinkaus, 1976; Shuster and Burgess, 2002; Danilchik et al., 2003; Kouranti et al., 2006; Boucrot and Kirchhausen, 2007; Dambournet et al., 2011; Giansanti et al., 2015; Figard et al., 2016). In the cleavage cycles that are present in many animal embryos, this need to rapidly supply membrane is especially apparent and is required to support the fast expansion and remodeling of furrow behaviors. Previous work on cleavage-stage embryos has shown that this membrane can come from the addition of new and/or recycled membrane by exocytic trafficking pathways or through the flattening of apical microvilli (Lecuit and Wieschaus, 2000; Shuster and Burgess, 2002; Danilchik et al., 2003; Holly et al., 2015; Figard et al., 2016; Mavor et al., 2016). Studies from an array of systems on the mechanisms of cytokinetic membrane has implicated exocytic and endocytic trafficking components, including SNARE proteins, the exocyst complex, the conserved oligomeric Golgi (COG) complex subunits, Clathrin, Arf-GEF and Rab proteins (Rab8, Rab11, Rab35 and more), as being required for furrow ingression (Low et al., 2003; Gromley et al., 2005; Farkas et al., 2003; Warner et al., 2006; Giansanti et al., 2007; Lee and Harris, 2013; Mavor et al., 2016; Dambournet et al., 2011; Kouranti et al., 2006). Thus, depending on cell requirements, it appears that cytokinetic membrane trafficking can occur through two major pathways: an endocytic recycling pathway and/or a new exocytic vesicles pathway. However, how these two pathways coordinate with each other in delivering furrow-associated vesicular membrane to ingressing furrows during cytokinesis is less understood.

Cleavage furrow formation during early *Drosophila melanogaster* embryogenesis offers a striking system to understand how membrane trafficking pathways drive furrow formation. As with many multicellular animal embryos, the first diploid nucleus is formed after fertilization, and then a series of rapid mitotic cycles occur. The resulting embryo undergoes nine rounds of syncytial divisions that occur deep within the yolk; however, during the tenth cell cycle, nuclei complete a bulk movement to the periphery of the embryo. This migration towards the embryonic cortex leads to a monolayer of nuclei that will subsequently undergo cellularization at cycle 14 to form the epithelial sheet, which will then gastrulate. As the nuclei begin to crowd into a common, subcortical layer, they initiate the formation of ingressing membrane furrows from the prospective apical surface of the cells. These furrows are rapidly formed in just a few minutes (2.5 mins at cycle 10 and ∼10 mins by cycle 13) and serve to separate and anchor individual mitotic figures before receding at the end of each syncytial cycle (cycles 10-13) (Sullivan and Theurkauf, 1995; Foe and Alberts, 1983; Holly et al., 2015; Xie and Blankenship, 2018; Xie et al., 2021). The four rounds of rapid furrow initiation, ingression and disassembly during the syncytial divisions makes it an appealing process to identify the pathways that promote deformation of the plasma membrane into deep furrowing processes (Grosshans et al., 2005; Lecuit and Wieschaus, 2000; Schejter and Wieschaus, 1993; Mazumdar and Mazumdar, 2002; Xie et al., 2018).

The Rab family of small GTPase proteins are major regulators of membrane trafficking networks. Individual Rab proteins are localized to distinct membrane compartments and are able to mediate the trafficking of vesicles from donor to acceptor compartments through their interactions with various effectors, which are often cargo, motor or tethering proteins (Pfeffer, 2005; Li and Marlin, 2015). Studies on specific Rab GTPase proteins have suggested key roles in directing furrow ingression in the early development of animal embryos. For example, disruption of the early endosomal Rab5 or the exocytic Rab8 slows or completely disrupts the rate of furrow ingression during *Drosophila* cellularization (Pelissier et al., 2003; De Renzis et al., 2002; Mavor et al., 2016). Other studies have also shown that Rab11 is required for efficient furrow ingression during *Drosophila* cellularization and in the *Caenorhabditis elegans* embryo, as well as in the meiotic cytokineses of *Drosophila* (Skop et al., 2004; Pelissier et al., 2003; Riggs et al., 2003; Giansanti et al., 2007). However, how membrane is sourced and recycled to potentiate furrow growth on rapid time scales in cleavage embryos is less clear. Here, we study the function of Rab39 and Rab35 in directing furrow ingression in the early syncytial cycles of the fly embryo. We find that Rab39 is present at a highly dynamic vesiculotubular network associated with the Golgi, and, in addition, these dynamics correlate with the growth of the rapid syncytial furrows in the early embryo. Disruption of *Rab39* leads to smaller, more dispersed Golgi structures and abolishes furrow formation, suggesting that Rab39 is essential for delivering membrane to the Golgi/trans-Golgi network (TGN), which is required for furrow ingression. We also find that Rab39 receives recycled endocytic membrane through a Rab35-, Rab4- and Klp98A-dependent pathway. Our data suggests an extensive endocytic pathway that operates in the early embryo and is essential for the recycling of membrane to the TGN for the initiation of the next cycle of furrow ingression in the cortical syncytium.

## RESULTS

### Dynamic TGN-associated Rab39 activity correlates with the onset of furrow ingression and is required for the maintenance of Golgi morphologies

**Fig. 1.**
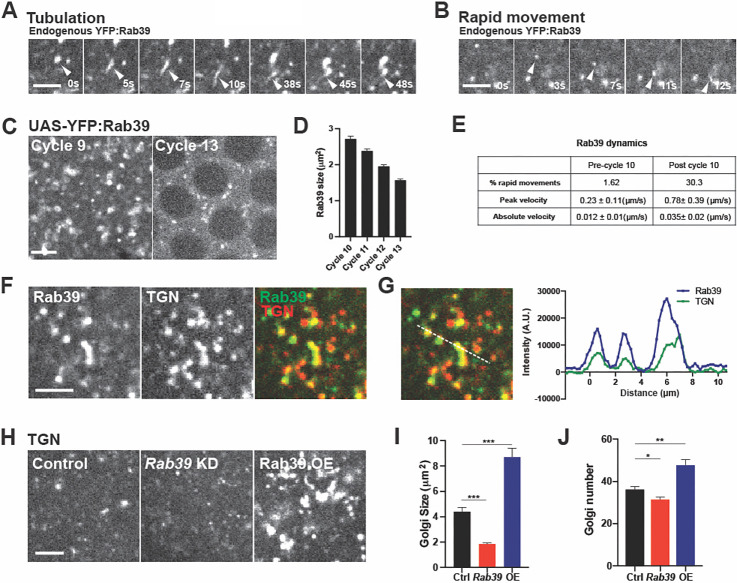
**Rab39 dynamics are enhanced at cycle 10 and are localized to the trans-Golgi network.** (A) Time points from live imaging of embryos endogenously expressing YFP:Rab39 showing the dynamics of tubulation at cycle 12. (B) Time points from live imaging of embryos endogenously expressing YFP:Rab39 showing rapid movements of Rab39 puncta at cycle 12. (C) Still images from embryos expressing UAS-YFP:Rab39 at cycle 9 and cycle 13. (D) Quantification of Rab39 compartmental size (UAS-YFP:Rab39) during the indicated cycles. (D,E) *n*=150 (cycle 10), 183 (cycle 11), 137 (cycle 12) and 120 (cycle 13) compartments. (E) Quantification of Rab39 dynamics at cycle 9 (pre-cycle 10) and cycle 11 (post cycle 10). Percentage of Rab39 compartments that display movements of 5 pixels (0.82 µm) or more in a 10 s period are indicated, along with peak and absolute velocities (see Materials and Methods). *n*=125 (pre-cycle 10) and 183 (post-cycle 10) Rab39 compartments measured. Error reported as standard deviations. (F) Still images from embryos expressing YFP:Rab39 and the trans-Golgi marker Galactosyltransferase:RFP under UAS control at cycle 12. (G) Intensity line plot of GalT:RFP and YFP:Rab39 shown in F across 10 µm distance. Dashed line indicates plotted line. (H) Still images from embryos expressing GalT:RFP in *Rab39* depleted (KD) or overexpression (OE) conditions at cycle 12. (I) Quantification of Golgi size in Rab39 shRNA or overexpression conditions in pixels. *n*=166 (Ctrl), 244 (*Rab39*) and 188 (OE) compartments in cell cycle 12. (J) Golgi compartmental densities found across the indicated conditions (Golgi number found in 100 µm^2^ region). *n*=9 (Ctrl), 10 (*Rab39*) and 10 (OE) measured embryos in cell cycle 12. *P<0.05; **P<0.005; ***P<0.0005 (Mann–Whitney *U*-test). Data are mean±s.e.m. Scale bars: 2 µm (A,B); 5 µm (C,F,H).

In previous work, we screened the *Drosophila* Rab family of proteins by either localization (Jewett et al., 2017) or function (Mavor et al., 2016) to identify the relevant membrane trafficking pathways that are active in the early embryo during morphogenesis. Among the Rab proteins that demonstrated punctate compartmental localization is Rab39 (Jewett et al., 2017). Rab39 is a lesser-studied Rab that shows remarkable dynamics in the early fly embryo (Fig. 1A,B) – we therefore focused on understanding how these behaviors contribute to development. Either UAS-YFP:Rab39 or endogenous YFP:Rab39 (YFP inserted at the endogenous *Rab39* genomic locus) showed intriguing behaviors. Rab39 compartments present in the early embryo before cycle 10 were large and relatively static structures; however, at cycle 10 they transitioned to becoming remarkably dynamic (Fig. 1C-E; Fig. S1A; Movie 1). Cycle 9 compartments were generally spherical in shape and could be as large as 3.5 µm^2^ in cross-sectional area before beginning to possess rapid movements and small tubulation events at cycle 10 (Fig. 1D,E; Movie 1). Rab39 compartments showed a nearly 20× increase in the number that displayed rapid movements at cycle 10, and peak velocities were observed to increase by ∼3-fold (Fig. 1E). Rab39 remained highly active through the remaining syncytial cycles (cycles 10-14). This onset of dynamic behaviors at cycle 10 was intriguing, as it was at cycle 10 that syncytial nuclei had migrated to the embryo periphery and began to organize rapid, cytokinetic-like plasma membrane furrows that serve to separate mitotic figures and provide spindle attachment points (Foe and Alberts, 1983; Sullivan et al., 1993; Holly et al., 2015; Xie and Blankenship, 2018). From cycle 10 through cycle 13, these plasma membrane furrows will undergo the formation and resolution of ever deeper transient furrows (Foe and Alberts, 1983; Sullivan et al., 1993; Holly et al., 2015; Xie et al., 2018). Interestingly, with each successive cycle of furrow formation, the size of Rab39 compartments became progressively depleted (Fig. 1C,D). Compartments at cycle 10 were 2.75 µm^2^ in average area, but were 42% smaller in area (1.60 µm^2^ average) by cycle 13. Thus, Rab39 shows dynamic behaviors and a depletion in compartmental size that correlates with the onset of syncytial furrow ingression.

These results would be consistent with Rab39 initiating dynamic behaviors that may be required for plasma membrane furrow formation – an early model could be that Rab39 is associated with a reservoir of membrane needed for furrow ingression. This led us to examine the localization of Rab39 compartments. Previous work has suggested that Rab39 is associated with Golgi in *Drosophila* S2 tissue culture cells or with late endosomes/secretory lysosomes in *Drosophila* neurons and tracheal cells (Chan et al., 2011; Gillingham et al., 2014; Caviglia et al., 2016). We therefore determined where Rab39 localizes in the early fly embryo. We found that the majority of Rab39 compartments localized to the Golgi apparatus, and, specifically, were present near the trans-facing tubular side of the Golgi [as marked by Galactosyltransferase:RFP (GalT:RFP)] (Fig. 1F,G; Fig. S1B,D; Movie 2) (Strous and Schachter, 1986). This result, and the vesiculotubular appearance of Rab39 during live imaging, suggest that Rab39 compartments are associated with the trans-Golgi network. To confirm this, we performed immunostaining to analyze the colocalization between Rab39 and cis- or trans-Golgi markers. Consistent with the previous results from live-imaging, Rab39 compartments were largely localized with trans-Golgi markers and adjacent to the cis-Golgi (88% of Rab39 compartments colocalized with GalT:RFP during live imaging and 56% colocalized with anti-Golgin245, a trans-Golgi marker, in fixed tissues) (Fig. S1B-D).

Given the dynamic Golgi localization of Rab39, we next explored whether the Golgi network requires Rab39 function. Indeed, compromising *Rab39* function revealed a deep disruption of the dispersed Golgi structures that are present in the *Drosophila* embryo (Fig. 1H). TGN size was strongly reduced in *Rab39*-disrupted embryos, as was the number of Golgi puncta (Fig. 1H-J). By contrast, overexpression of Rab39 induced the formation of significantly larger Golgi compartments (Fig. 1H-J). These results demonstrate that Rab39 is required for Golgi apparatus morphologies and may direct the transport of membrane and proteins from other organelles to the TGN.

### Disruption of *Rab39* leads to defects in cleavage furrow ingression

**Fig. 2.**
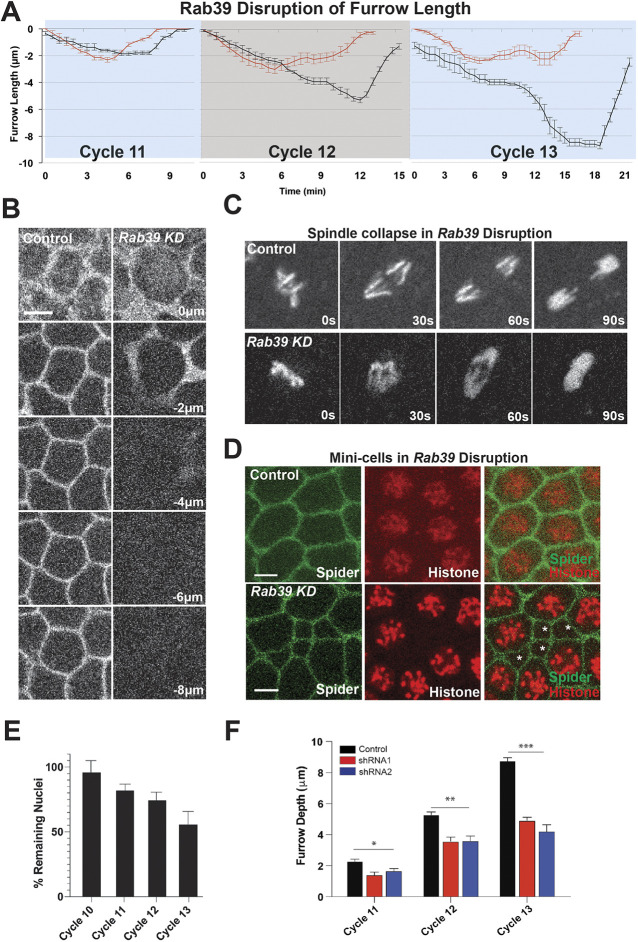
**Disruption of *Rab39* function causes furrow defects.** (A) Furrow dynamics in control (black) and *Rab39* shRNA (red) embryos during syncytial cycles 11-13. *Rab39*: *n*=15 (cycle 11), 18 (cycle 12) and 15 (cycle 13) measurements; control: *n*=15 (cycle 11), 15 (cycle 12) and 12 (cycle 13) measurements (three measurements/embryo). (B) Maximal furrow depths at cycle 13 in control and *Rab39* knockdown embryos (Resille:GFP plasma membrane marker). *z*=0 µm is the apical surface and *z*=−8 µm is the most basal. (C) Still images showing mitotic division failure based on spindle collapse in embryos expressing *Rab39* shRNA and Histone:RFP compared with control. Images from cycle 11 embryos. (D) Still images from live imaging of plasma membrane (Spider:GFP) and Histone:RFP showing abnormalities in apical furrow dimensions resulting from nuclear fallout compared with control. Images from cell cycle 11 embryos. Asterisks show mini-cell phenotype. (E) Percentage of remaining nuclei in *Rab39* knockdown embryos after each mitotic cycle (see Materials and Methods). (F) Maximal furrow depths in control and two independent Rab39 shRNA lines. *n*=20 measurements from at least five embryos. *P<0.05; **P<0.005; ***P<0.0005 (Mann–Whitney *U*-test). Data are mean±s.e.m. Scale bars: 5 µm.

Given the timing of the onset of rapid Rab39 dynamics, we asked whether Rab39 function is required for plasma membrane ingression and syncytial furrow formation. To do so, we imaged embryos expressing a plasma membrane marker (Resille:GFP) after *Rab39* disruption (phenotypes were confirmed with two independent Rab39 shRNAs and CRISPR-based gRNA expression; Fig. 2F; Fig. S2). These data revealed that Rab39 is essential for the formation of deep, syncytial furrows (Fig. 2A,B,F; Fig. S2A,E). Membrane furrows in control embryos ingressed ∼2-3 µm deeper with each successive cycle, until by cycle 13 they reached ∼8 µm in depth. This was through a biphasic process that consisted of an initial ingression phase (Ingression I), which was followed by stabilization of furrow lengths, and then a further rapid zygotically-driven ingression phase (Ingression II; Fig. 2A,B,F; Xie et al., 2018). Interestingly, in *Rab39*-disrupted embryos the membrane furrows in each cycle lengthened only to ∼2 µm before receding. This made the difference most apparent in cycles 12 and 13, where Ingression II appeared to be nearly abolished (Fig. 2A,B). Indeed, furrow lengths were reduced by 78% at cycle 13, whereas maximal furrow ingression rates were only 0.33 µm/min in *Rab39*-disrupted embryos compared with 1.2 µm/min in control embryos. Furrows also showed the broadened morphologies typically seen when Ingression II is disrupted (Fig. 2B; Xie and Blankenship, 2018).

The formation of the syncytial furrows is required to properly anchor and corral mitotic spindles (Holly et al., 2015), and *Rab39* embryos showed a deep disruption in successful mitoses (Fig. 2C,D; Fig. S2B-D). Mitotic spindles were observed to collapse as they attempted to achieve anaphase separation (Fig. 2C) and the resultant polyploid nuclei fell away from the embryonic cortex into the yolk, causing cell boundaries to shrink in size, creating ‘mini-cells’ (Fig. 2D). This high level of mitotic defects and subsequent nuclear fallout resulted in fewer nuclei populating the embryo cortex. By cycle 13, only 56% of nuclei observed in controls remained in the plane of nuclei at the embryo periphery (Fig. 2D,E). The deep defects in furrow ingression observed after reduction of Rab39 function suggest that it is a key regulator of the membrane trafficking pathways that are required for cytokinetic-like furrow formation in the early embryo.

### Rab39 tubulation and displacement dynamics are microtubule dependent

**Fig. 3.**
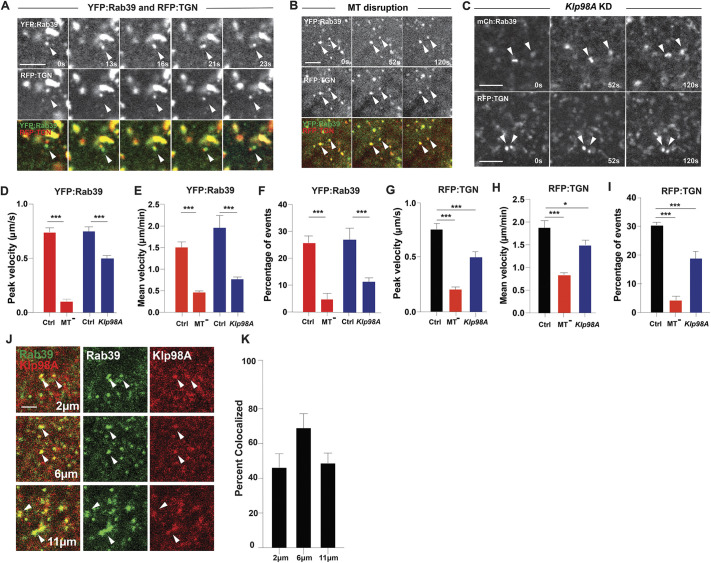
**Rab39/Golgi transport is microtubule and Klp98A motor dependent.** (A,B) Time points from live imaging of endogenously expressed YFP:Rab39 and TGN:RFP (GalT:RFP) showing the movement of Rab39 and TGN compartments in control (A) and colchicine-injected (B) embryos. Arrowheads mark representative compartments. (C) Time-lapse images of embryos expressing mCh:Rab39 or GalT:RFP with *Klp98A* shRNA. Arrowheads mark representative compartments. (D) Peak velocity of TGN compartments in control [water-injected (red, *n*=122) or Gal4-only siblings (blue, *n*=150)], colchicine-injected (MT^−^; *n*=106) and *Klp98A* shRNA (*n*=100) embryos. (E) Mean velocity of TGN compartments in control [water-injected (red) or Gal4-only siblings (blue)], colchicine-injected and *Klp98A* shRNA embryos. (F) Percentage of TGN compartments (GalT:RFP) that displayed rapid movement [movements of 5 pixels (0.82 µm) or more over a 10 s period] in control [water-injected (red) or Gal4-only siblings (blue)], colchicine-injected and *Klp98A* shRNA embryos. (G) Peak velocity of Rab39 compartments in control (GalT:RFP), colchicine-injected and *Klp98A* shRNA embryos. (H) Mean velocity of Rab39 compartments in control (GalT:RFP), colchicine-injected and *Klp98A* shRNA embryos. (I) Percentage of Rab39 compartments that displayed rapid movement in control (GalT:RFP), colchicine-injected and *Klp98A* shRNA embryos. *n*=145(Ctrl), 138 (Colch) and 124 (*Klp98A*) compartments in G-I. (J) Fixed embryos expressing endogenous YFP:Rab39 stained with anti-GFP and anti-Klp98A antibodies. Arrowheads mark representative colocalized compartments. (K) Percentage Rab39 puncta colocalized with Klp98A puncta at 2, 6 and 11 µm below the apical surface (see Materials and Methods). *n*=95 (2 µm), *n*=92 (6 µm) and *n*=111 (11 µm) puncta. Data from cycle 12 embryos. *P<0.05; ***P<0.0005 (Mann–Whitney *U*-test). Data are mean±s.e.m. Scale bars: 5 µm, except in J (2 µm).

Given the potential role of Rab39 in directing trafficking from other organelles to the Golgi, we next examined what drives the rapid changes in Rab39 behaviors observed during live imaging. These analyses may also provide insights into how the TGN is shaped in the early embryo. Imaging embryos expressing YFP:Rab39 and GalT:RFP (Fig. 3A) revealed that Rab39 compartments were highly motile and dynamic, with a mean velocity of 1.5 μm/min (Fig. 3E). We also noticed a subpopulation of Rab39 compartments that moved very rapidly, with peak velocities of 0.7 μm/s (Fig. 3D,F). During these ‘active’ movements, brief associations were often observed between Rab39 and the trans-Golgi network (Fig. 3A; Movie 2). It is interesting to note that some Golgi compartments also moved actively and displayed similar motility to Rab39, with a mean velocity of 1.8 μm/min and a peak velocity of 0.75μm/s (Fig. 3G,H). The rapid movements and associations with the TGN, combined with the data showing that alterations in Rab39 function can lead to dramatic changes in Golgi size, suggests that Rab39 compartmental function directs active transport to the Golgi.

Given the observed dynamics, we wanted to know whether these movements are microtubule-dependent, which would also suggest the involvement of microtubule motor proteins. Indeed, inhibiting the formation of microtubules by acute colchicine treatment led to a decrease in the motility of Rab39 as well as the frequency of rapid movements (Fig. 3B,D-F). Rab39 compartments also became less tubular and more punctate in appearance (Fig. 3B). TGN-like compartments, as marked by GalT:RFP, also showed a reduction in motility (Fig. 3G-I). These results suggest that Rab39-mediated transport is microtubule dependent, and prompted us to examine whether we could identify a motor protein involved in Rab39 function and the formation of a compartmental pool that fuels plasma membrane furrow ingression.

### A Kinesin-3 family member, Klp98A, directs Rab39-Golgi trafficking along microtubules

Systematic studies in *Drosophila* using Rab affinity chromatography have identified several potential proteins that interact with Rab39 (Gillingham et al., 2014). These include the Kinesin-3 family motor protein Unc-104, although this interaction has not been further studied outside of these Rab affinity screens (Gillingham et al., 2014). We first tested whether Unc-104 might mediate the movements of Rab39 by imaging an Unc-104:GFP construct as well as disrupting the function of Unc-104. We found that Unc-104:GFP demonstrated no punctate localization in the early embryo, nor did functional disruption of Unc-104 (shRNA-mediated disruption) affect early development (data not shown). As Unc-104 did not appear to be a good candidate to mediate Rab39 activities, we examined the other Kinesin-3 family members present in the *Drosophila* genome, Kin-73 (Khc-73) and Klp98A. Only Klp98A showed a strong localization and functional requirement in the early embryo. Indeed, immunohistochemistry against Klp98A and YFP:Rab39 demonstrated that Klp98A was also present in compartmental-like puncta in the early embryo (Fig. 3J,K). Further, Klp98A and Rab39 possessed a significant degree of colocalization. In cycle 12 embryos, ∼52% of Rab39 compartments were colocalized with Klp98A puncta (Fig. 3K), with the highest degree of colocalization found ∼6 µm into the embryo, as measured from the surface of the embryo (Fig. 3K). This result is intriguing, as furrows ingress to a maximum length of ∼6 µm in cycle 12 (Xie and Blankenship, 2018), suggesting that Klp98A could help target the delivery of Rab39/Golgi-dependent membrane pools to a region near ingressing furrows.

We then examined whether Rab39 compartmental behaviors and/or TGN movement requires Klp98A function. Disruption of *Klp98A* with Klp98A shRNA led to a decrease in rapid movements of Rab39 and TGN-associated compartments (Fig. 3C-F). The peak and mean velocities of Rab39 compartments were significantly reduced in *Klp98A* compromised embryos (0.53 µm/s peak velocities and 0.72 µm/min mean velocity, a 38% and 65% reduction from control measurements, respectively) (Fig. 3D,E). In addition, TGN punctate compartments showed a similar reduction in dynamics (Fig. 3C,G-I). These results suggest that Klp98A functions as a microtubule motor protein directing Rab39-Golgi transport.

Lastly, we explored whether Rab39 is required for the proper recruitment and localization of Klp98A to compartmental structures. If Rab39 is responsible for associating the Klp98A motor to the membrane compartment, it would be expected that Klp98A localization is altered after Rab39 disruption. Indeed, a striking difference in Klp98A localization was observed in *Rab39*-disrupted embryos. The Klp98A compartmental puncta seen in control embryos were lost after Rab39 function was compromised. Instead, Klp98A localized to large aggregates located near the nucleus (Fig. S3A,B). Staining for DNA and the nuclear envelope (with DAPI and anti-Lamin, respectively) revealed that Klp98A was mislocalized to large cytoplasmic chromosomal fragments. These fragments likely arise during mitotic failures caused by furrow defects in these embryos (see below). These results suggest that Rab39 and Klp98A function together to control the transport to and localization of TGN-associated membrane compartments.

### Klp98A is required for furrow formation during syncytial division

**Fig. 4.**
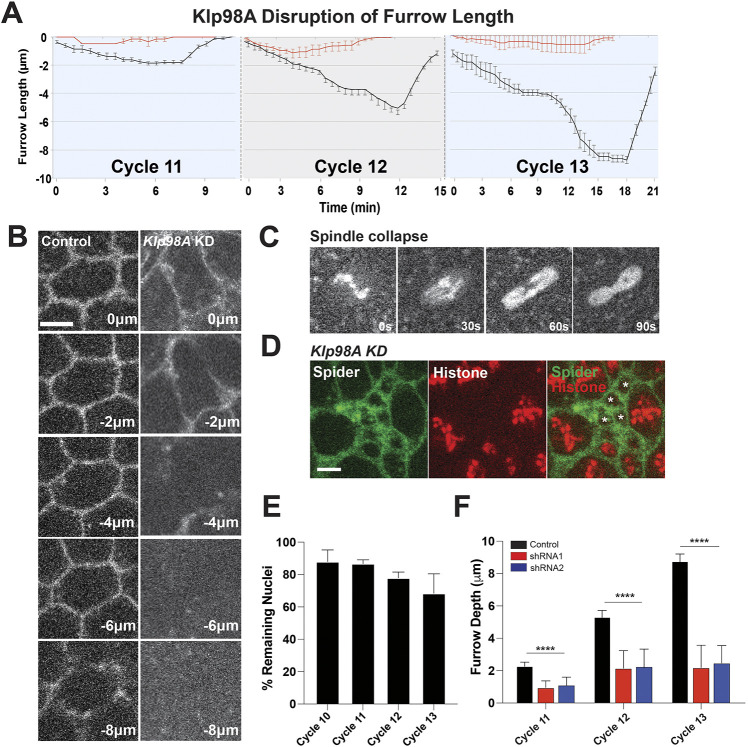
***Klp98A* disruption produces similar defects in furrow formation.** (A) Furrow length measurements over time of control (black) and *Klp98A* shRNA (red) embryos from syncytial cycles 11-13. Control: *n*=15 (cycle 11), 15 (cycle 12) and 21 (cycle 13); *Klp98A*: *n*=9 (cycle 11), 18 (cycle 12) and 12 (cycle 13) measurements (three measurements/embryo). (B) Still images from live imaging of control and *Klp98A*-disrupted embryo expressing Spider:GFP (plasma membrane marker) showing maximal furrow length depth during cycle 13. *z*=0 µm is the apical surface and *z*=−8 µm is the most basal. (C) Still images of His-2av:RFP showing mitotic division failure at cycle 11 in *Klp98A* embryo from spindle collapse. (D) Still images of mini-cell and furrow abnormalities at cycle 12 caused by nuclear fallout. Asterisks show minicell phenotype. (E) Analysis of nuclear fallout based on percentage of nuclei remaining. (F) Maximal furrow depth measurements in *Klp98A* embryos confirmed with two independent shRNAs. *n*=25 measurements from at least four embryos each. ****P<0.00005 (Mann–Whitney *U*-test). Data are mean±s.e.m. Scale bars: 5 µm.

Given the observed similarities between Rab39 and Klp98A, we examined whether furrow formation was also compromised in *Klp98A*-disrupted embryos. Disrupting Klp98A function resulted in deeply affected furrows, which reached a maximum depth of ∼1.6 µm (Fig. 4A,B,F). In addition to furrow defects, resultant mitotic defects showed collapsed spindles leading to a similar nuclear fallout and ‘mini-cell’ phenotype to that observed in *Rab39*-disrupted embryos (Fig. 4C,D). By cycle 13, only 68% of nuclei remained near the embryonic cortex (Fig. 4E). The similar phenotypes seen with *Klp98A* disruption suggest that this motor protein is also essential for membrane trafficking during furrow formation and may be involved in the same furrow-driving pathway as Rab39.

### A screen of Rab compartmental behaviors suggests that Rab39 receives membrane stores from Rab35/Rab4-mediated trafficking pathways

**Fig. 5.**
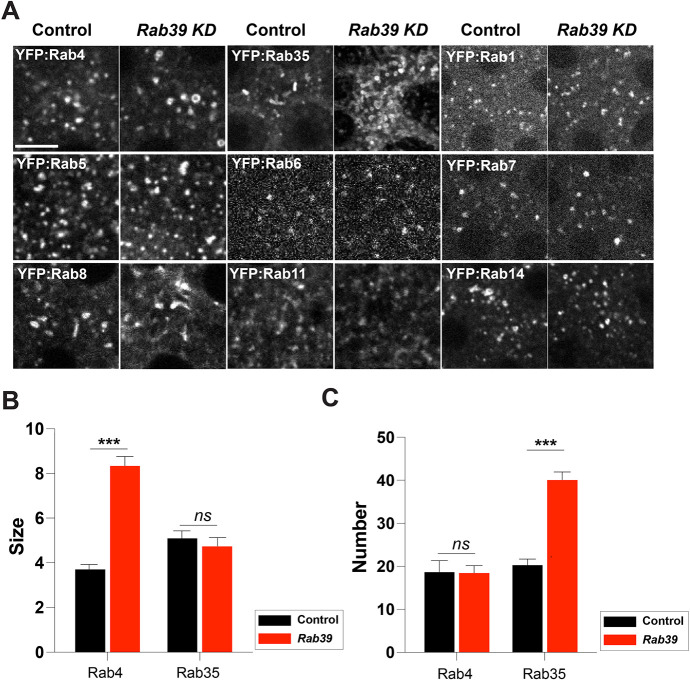
**A live-imaging based screen of Rab proteins reveals Rab35 and Rab4 enlargement in *Rab39*-compromised embryos.** (A) Still frames from live imaging of embryos expressing YFP-labeled Rab proteins in control or *Rab39* shRNA embryos. (B) Quantification of Rab4 or Rab35 size (µm^2^) in control and *Rab39*-disrupted embryos. *n*=100 (Rab4, control), 102 (Rab4, *Rab39*-disrupted), 109 (Rab35, control) and 121 (Rab35, *Rab39*-disrupted) compartments. (C) Number of Rab4 or Rab35 compartments in 100 µm^2^ region in control and *Rab39*-disrupted embryos. *n*=18 (Rab4, control), 15 (Rab4, *Rab39*-disrupted), 42 (Rab35, control) and 21 (Rab35, *Rab39*-disrupted) regions measured (three regions measured/embryo). Data from cycle 12 embryos. ***P<0.0005 (Mann–Whitney *U*-test). ns, not significant. Data are mean±s.e.m. Scale bar: 5 µm.

As Rab39 is required for trafficking to the Golgi during furrow formation, we explored where the membrane reservoir for this pathway may be derived from – in theory, a variety of possible pathways could deliver via the Rab39 pathway. These include pathways of newly synthesized cargo from the endoplasmic reticulum (ER) and cis-/medial-Golgi, or recycled membrane stores that may originate from remodeling of the cell surface. As an initial starting point to uncover the source compartment, we performed live-cell imaging to screen the localization of nine different Rab family proteins that earlier work has shown are present, to varying degrees, in the embryo at these stages (Zhang et al., 2007; Mavor et al., 2016; Jewett et al., 2017; Fig. 5A). We compared the behaviors of these Rab proteins between control and *Rab39* shRNA embryos and found that, of the screened Rab family proteins, Rab35 and Rab4 had the deepest alterations in compartmental sizes after Rab39 disruption. Of note, Rab35 and Rab4 compartments appeared to accumulate in cells, growing larger in size and/or becoming more numerous (Fig. 5A-C). Rab35 compartments more than doubled in number, whereas Rab4 compartments grew 2.4-fold in size (Fig. 5B,C). Indeed, the changes in Rab35 compartments were particularly striking, with apical regions filled with numerous Rab35 tubular and compartmental structures (Fig. 5A). These results would fit with a model in which Rab39 disruption leads to a failure in delivering membrane cargo from Rab35/Rab4 to the TGN/Rab39 compartments, with the resultant accumulation of Rab35/Rab4 compartments. As Rab35 and Rab4 are classically identified as endosomal Rab proteins, these results suggest Rab39 may direct the movement of endocytically recycled plasma membrane cargo from Rab35/Rab4 pathways to the TGN.

### Rab39 receives membrane stores recycled through a Rab35-mediated endocytic pathway

**Fig. 6.**
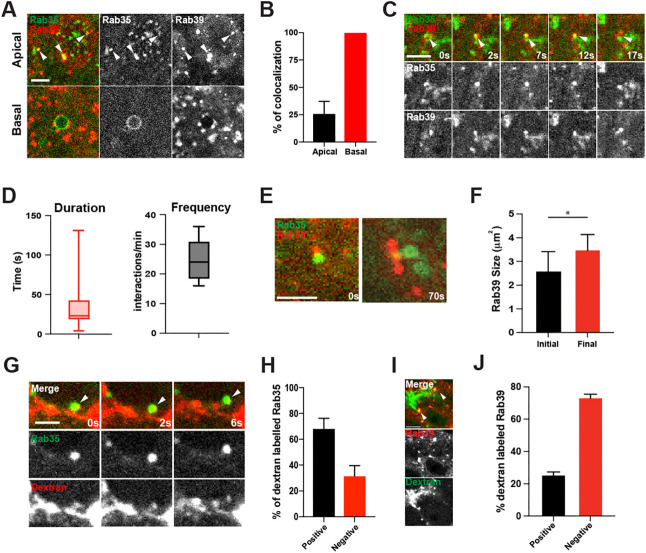
**A Rab35-mediated endocytic pathway delivers recycled plasma membrane to Rab39 compartments.** (A) Still frames from live imaging of embryos expressing CRISPR GFP:Rab35; mCherry:Rab39. Arrowheads mark the colocalized Rab35 and Rab39 compartments. (B) Percentage of colocalization between Rab39 and Rab35. *n*=12 regions measured from four embryos (see Materials and Methods). (C) Images of the association between CRISPR GFP:Rab35 and mCherry:Rab39 over time. Arrowheads mark the associated Rab35 and Rab39 compartments. (D) Quantitation of duration and frequency of Rab35/Rab39 interactions. Box plot shows the 25th quartile (bottom of the box), the median (middle of the box) and 75% quartile (top of the box); the whiskers represent the minimum (below the box) and the maximum (above the box) values. (E) Still images from live imaging of GFP:Rab35 and mCherry:Rab39 during a prolonged interaction. (F) Quantitation of Rab39 puncta size at beginning and end of an interaction with Rab35. Interactions selected were >60 s. *n*=10. (G) Time points from live imaging of embryos expressing CRISPR GFP:Rab35 injected with dextran. Arrowheads show the compartment that acquires both Rab35 and dextran signals. (H) Percentage of dextran-labeled Rab35 compartments. *n*=15 regions measured from five embryos (see Materials and Methods). (I) Still images from live imaging of dextran-injected embryos expressing mCherry:Rab39. Arrowheads show the compartment with Rab39 and dextran colocalization. (J) Percentage of Rab39 puncta labeled with dextran in injected embryos. *n*=15 embryos. Data from cycle 12 embryos. *P<0.05 (Mann–Whitney *U*-test). Data are mean±s.e.m. Scale bars: 2 µm (A,C,G,I); 2.5 µm (E).

As Rab35 displayed the most striking changes after disruption of *Rab39*, we next examined the interaction between Rab35 and Rab39. We first determined the extent of colocalization between Rab35 and Rab39. About 25% of Rab35 compartments also colocalized with Rab39 in apical regions (0-4 μm below the apical surface); however, 100% of basal compartments had Rab39 puncta associated with them (Fig. 6A,B). Notably, the extent of overlap is not fully coincident, but instead appeared to be consistent with two compartments (a Rab35 and a Rab39 compartment) that are tightly juxtaposed. Time-lapse imaging of apical CRISPR GFP:Rab35 and mCh:Rab39 revealed that these compartments often had transient associations. Rab39 compartments were frequently observed to move adjacent to Rab35 compartments, and then appear to be docked, associating with the Rab35 puncta for ∼25 s on average (Fig. 6C,D). However, this period was highly variable and some associations lasted for longer than 120 s (Fig. 6D,E; Movie 3). Interestingly, in longer-lived associations, Rab39 compartments visibly enlarged, growing by greater than 40% in cross-sectional area before then dissociating from the Rab35 compartment (Fig. 6E,F). These results further suggest that Rab39 may receive membrane cargo from a Rab35-mediated pathway. As Rab35 has been implicated in recycling plasma membrane through endocytic pathways (Kouranti et al., 2006; Jewett et al., 2017; Miao et al., 2019), we asked whether the delivery from Rab35 to Rab39 was carrying endocytically-derived cargo. To test this, we tracked the dynamics of CRISPR GFP:Rab35 and Alexa Fluor 568-dextran following dextran injection into the perivitelline (extracellular) space of the embryo. We found that 64% of Rab35 compartments were colocalized with labeled dextran immediately after injection (Fig. 6G,H), thus likely representing endocytic uptake from the plasma membrane and consistent with previous results from gastrulation stages of the embryo (Miao et al., 2019). *Rab35* disruption also reduced the number of dextran-positive cytoplasmic puncta at these stages (Fig. S4A,B). Rab39 compartments were also observed to contain extracellularly-derived dextran, although at lower frequencies (Fig. 6I,J). These results suggest that recycled cell surface-derived membrane cargo may be delivered to Rab39 compartments through a Rab35-mediated endocytic pathway.

**Fig. 7.**
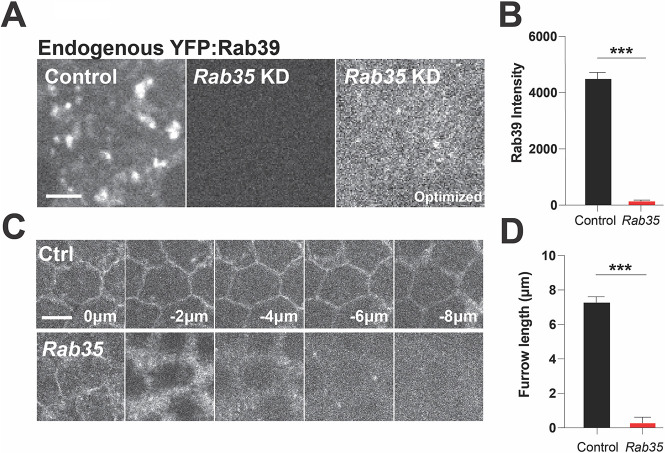
**Loss of Rab35 function results in depletion of Rab39 compartments and defective furrow ingression.** (A) Still frames from live imaging of embryos expressing endogenous YFP:Rab39 in control, *Rab35* shRNA (identical imaging settings as control) and *Rab35* shRNA optimized imaging conditions (leveled to see remaining signal) in cycle 12 embryos. (B) Quantitation of YFP:Rab39 intensity (*n*=23 puncta). (C) Still frames from live imaging of plasma membrane marker (Spider:GFP) in control and *Rab35* shRNA conditions during cycle 13. *z*=0 µm is the apical surface and *z*=−8 µm is the most basal. (D) Quantitation of maximal furrow length in cycle 13 of control and *Rab35*-disrupted embryos (*CRISPR GFP:Rab35* embryos expressing deGradFP) (*n*=15 regions measured in five embryos). ***P<0.0005 (Mann–Whitney *U*-test). Scale bars: 5 µm.

If Rab39 receives membrane cargo from a Rab35-dependent pathway, one expectation would be that disruption of Rab35 should lead to defects in Rab39, with potentially smaller and/or less numerous Rab39 compartments being observed. We thus imaged endogenous YFP:Rab39 in *Rab35* compromised embryos and found that there was a deep loss of Rab39 compartments and a 95% decrease in Rab39 fluorescence intensity in these embryos (Fig. 7A,B). This would be consistent with a model in which Rab35 delivers cargo to Rab39 compartments. The reduction in Rab39 compartments was also large enough that the examination of dextran-labeling of these compartments after Rab35 disruption was not possible. Interestingly, Rab39 compartments also had reduced size after *Rab4* disruption, although this was a moderate change in Rab39 behaviors compared with *Rab35* disruption (Fig. S5A). However, these combined data were again consistent with both Rab4 and Rab35 acting upstream of Rab39. Unlike Rab39, Rab35 behaviors appear to be relatively unchanged after *Rab4* disruption (Fig. S5B), whereas Rab4 compartments were much larger in size, but fewer in number, after *Rab35* disruption (Fig. S5C). Similar to Rab35 and Rab39, Rab4 compartments also fill with dextran, consistent with their participation in an endocytic pathway (Fig. S6A,B). Lastly, we examined whether plasma membrane furrow ingression during the syncytial cycles was dependent on *Rab35* function. To do so, we made use of a deGradFP-mediated disruption of *CRISPR GFP:Rab35* function (deGradFP is a GFP nanobody system that targets GFP-labeled proteins for degradation; Caussinus et al., 2011). Homozygous *CRISPR GFP:Rab35* embryos that expressed deGradFP possessed deep failures in furrow ingression, with furrows reaching only 0.5 µm in depth at cycle 13 (Fig. 7C,D). This phenotype is similar to what was observed in both *Rab39*- and *Klp98A*-disrupted embryos, and again supports a model in which Rab35 is involved in a common membrane trafficking pathway with Rab39 and Klp98A to recycle plasma membrane to the TGN for eventual use during furrow ingression.

## DISCUSSION

**Fig. 8.**
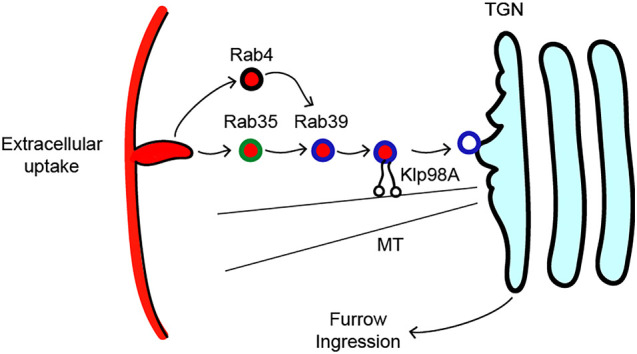
**Model for membrane recycling to the Golgi by Rab35, Rab39 and Klp98A.** Our model proposes that Rab35 and Rab4 are involved in endocytic uptake of plasma membrane during the syncytial stage of embryogenesis, which is necessary for the remodeling of subsequent furrow behaviors. Rab35 and Rab4 send membrane to the trans-Golgi network through Rab39, which is transported and localized via Klp98A. This process is microtubule (MT) dependent and is a recycling pathway that is necessary for Golgi-mediated furrow ingression.

Our studies reveal an endocytic recycling pathway that targets membrane-to-Golgi compartments to permit the rapid formation of cleavage furrows in the early *Drosophila* embryo (Fig. 8). We find that Rab39 localizes to a dynamic vesicular and tubular compartment that is positive for TGN and trans-Golgi markers. A Kinesin-3 family member, Klp98A, is required for the observed rapid dynamics of Rab39 compartments, as well as the proper distribution of Rab39 compartments. Disruption of Rab39 or Klp98A leads to a deep failure in furrow ingression, with furrows reaching a maximum depth of ∼2 µm in compromised embryos. Dextran labeling to mark endocytic uptake during furrow formation demonstrates that Rab39 receives cargo from Rab35 compartments, consistent with previous work showing that Rab35 serves to drive the efficient uptake of plasma membrane components, and Rab35 disruption also leads to a failure in furrow ingression. Interestingly, Rab35 and Rab39 compartments are often found in close proximity and display dynamic associations in apical regions near the plasma membrane. Thus, our data demonstrate a Rab39-Klp98A-Rab35 pathway that is necessary for furrow ingression in the fly embryo.

Many multicellular animal embryos possess rapid cleavage stages in which there is a requirement for the mobilization of cytoskeletal and membrane trafficking networks to enable plasma membrane ingression in a timescale in the order of minutes. This is likely due to the rapid expansion of cell surface area that occurs during these stages as embryos double their cell and/or nuclei number with each round of division. However, it is not only during embryonic cleavage cycles that membrane trafficking is a key requirement for cell division. For example, in the *Drosophila* male germline, membrane addition is essential to enable the cell-shape changes that drive cell division, as both elongation of the cell at anaphase as well as the ingression of the cytokinetic furrow depending on membrane recycling and addition pathways (Xu et al., 2002; Dyer et al., 2007; Giansanti et al., 2007, 2015). Many differentiated cell types also depend on membrane trafficking during cytokinetic abscission (reviewed by Albertson et al., 2005; Montagnac et al., 2008; Schiel and Prekeris, 2013; Frémonet and Echard, 2018). Indeed, a host of endosomal Rab proteins (Rab4, Rab5, Rab8, Rab11, Rab14, Rab21 and Rab35) have been implicated, to varying degrees, in contributing to cytokinetic processes, with Rab11 and Rab35 having the best validated roles (reviewed by Gibieža and Prekeris, 2018). It is interesting that Rab35 and Rab4 also appeared through our screening of Rab family proteins that affect Rab39 compartmental behaviors, whereas Rab11 has previously been implicated in furrow formation during cellularization in the fly embryos (see below; Pelissier et al., 2003). In addition, previous work from our lab has shown that Rab8 directs the final exocytic delivery of membrane required for furrow ingression (Mavor et al., 2016), but does not appear to be associated with the earlier endocytic pathways described in this study. Although different cell types may use distinct Rab proteins, and may have different sensitivities to disruptions of membrane trafficking pathways, it appears that the endocytic recycling and redelivery of membrane to the cell surface to drive the topological changes necessary for furrowing processes is often a common theme.

Our data have shown that two Rab proteins, Rab35 and Rab39, cooperate in the membrane-dependent remodeling of the cell surface that drives furrow ingression. As mentioned above, the involvement of Rab35 is interesting, as it has been previously implicated in cell division and abscission (Kouranti et al., 2006; Dambournet et al., 2011; Frémont et al., 2017). Rab35 also provides an intriguing bridging function between trafficking networks and the remodeling of polyphosphatidylinositols (PIPs) and F-actin (Dambournet et al., 2011; Frémont et al., 2017; Miao et al., 2021), and previous work during *Drosophila* gastrulation has shown that Rab35 acts as endocytic efficiency platforms, where it functions at tubular plasma membrane invaginations to direct rapid endocytic events (Jewett et al., 2017; Miao et al., 2019). Rab35 can also influence (and be influenced by) actomyosin network function (Jewett et al., 2017). Although Myosin II function appears to be dispensable for furrow ingression at these stages (Royou et al., 2004), recent work has shown that Myosin is necessary for the bending of actin cap structures into incipient furrows (Zhang et al., 2018), and it will be interesting to examine in future work whether Rab35 endocytic tubules pose a convergence point between F-actin, PIPs and trafficking networks during furrow ingression in the early embryo. In addition to the impact of Rab35 on Rab39, Rab39 compartment size, but not number, depends on Rab4 function. Where Rab4 may function in this pathway relative to Rab35 is less clear. Rab35 possesses both plasma membrane and cytoplasmic compartmental distributions, whereas Rab4 is only present as compartments within the apical cytoplasm. Unlike Rab39, Rab35 behaviors appear to be relatively unchanged after *Rab4* disruption, whereas Rab4 compartments are much larger in size, but fewer in number, after *Rab35* disruption. Rab4 is canonically associated with early endosomal function, so it may be that Rab4 impacts Rab35-Rab39 trafficking through its role as an intermediate in the endocytic delivery of membrane stores to Rab39 and the TGN.

Rab39, on the other hand, has been found associated with Golgi, late endosomal or secretory lysosomal structures (Chen et al., 2003; Chan et al., 2011; Seto et al., 2011; Gillingham et al., 2014; Caviglia et al., 2016). Although Rab39 localization may vary, Rab39 compartmental function often appears to be engaged and highly dynamic when cells need to grow particular surfaces. Our work demonstrates that Rab39 is required for rapid furrow ingression; however, other examples of Rab39 function in *Drosophila* are when adjacent tubular networks of the *Drosophila* tracheal system anastomose to create a continuous lumen. Here, tracheal cells mobilize Rab39 compartments that contain markers of secretory lysosomes to directly drive budding of the tracheal surface and fusion of neighboring lumens (Caviglia et al., 2016). By contrast, in the early embryo it appears that Rab39 stores are likely bridged by Rab8 exocytic compartments to provide the targeted addition necessary for furrow ingression (Mavor et al., 2016). In addition to Rab8, the exocyst complex and RalA also function in directing the final targeting of membrane stores to the furrow (Holly et al., 2015). Foundational work in the early embryo has shown that the golgin Lava Lamp (Sisson et al., 2000) and the recycling endosomal protein Rab11 (Pelissier et al., 2003) are involved in this membrane addition pathway. It is interesting that Rab11 did not appear to affect Rab39 in our screening of Rab family proteins – this would be consistent with Rab11 functioning in a secretory portion of the furrow-building process or, possibly, through a parallel pathway to the Rab39-Rab35-Klp98A pathway. We would also note that while this study demonstrates the fundamental importance of the recycling Rab39/Rab35 pathway to furrow ingression, it also does not address the relative contributions of this recycling pathway versus those of other potential membrane sources (such as newly synthesized cargo). Although it is clear that our understanding of the trafficking pathways that direct early morphogenesis has advanced considerably in the last few years, it is also clear that much more remains to be discovered.

## MATERIALS AND METHODS

### Fly stock and genetics

The following fly stocks were used in this study: His2Av:RFP (23650), His2Av:RFP (23651), endogenous YFP:Rab39 (62560), UAS-Rab39 Valium 22 (51689), UAS-Rab39 Valium 20 (53995), UAS-YFP:Rab39 (9835), UAS-Klp98A Valium20 (39037), UAS-GalT:RFP (65251), endogenous YFP:Rab1 (62539), UAS-YFP:Rab5 (24616), UAS-YFP:Rab11 (9790), UAS-YFP:Rab4 (23269), UAS-YFP:Rab6 (23251), endogenous YFP:Rab7 (62545), UAS-YFP:Rab8 (9782), UAS-YFP:Rab14 (9794), UAS-Rab4 Valium 20 (33757), UAS-Unc-104 Valium 22 (43264), UAS-Unc-104 Valium 20 (53296), UAS-Kin-73 Valium 22 (38191), UAS-Kin-73 Valium 20 (36733) (all from the Bloomington *Drosophila* Stock Center); CRISPR GFP:Rab35 (Jewett et al., 2017); UAS-Unc-104:GFP (Barkus et al., 2008); Resille:GFP (Morin et al., 2001; Blankenship et al., 2006); Spider:GFP (Morin et al., 2001; Blankenship et al., 2006). To generate Rab39 Walium 22 and Klp98A Walium, primers were designed using the protocol described by the Drosophila Research and Screening Center (https://fgr.hms.harvard.edu/knockdown-vectors). Primers were annealed and cloned into pWALIUM22 plasmid (DSRC, https://fgr.hms.harvard.edu/trip-plasmid-vector-sets). To generate UAS-mCherry:Rab39, N-terminal mCherry was inserted into pUASp along with the Rab39 coding sequence. All DNA constructs were validated by DNA-sequencing. Valium and Walium constructs are bioinformatically validated against off-target binding and further validated through the use of independent shRNA lines that produce similar defects. Finally, the common phenotypes observed in CRISPR gRNA and separate shRNA lines demonstrates the specificity of these interventions. Neutral RNAi (Rhodopsin 3) or deGradFP does not disrupt development at these stages (Mavor et al., 2016; Jewett et al., 2017). Embryos were collected from cages at 25°C, except embryos from Valium lines and Walium lines, which were collected at 18°C. UAS transgenic flies were crossed to matαTub-Gal4VP16 67C;15 (D. St Johnson, Gurdon Institute, Cambridge, UK) maternal driver females.

### Live imaging and injection

Embryos were dechorionated in 50% bleach solution for 2 min and then washed with water. Embryos were then relocated to a slide with a gas-permeable membrane in Halocarbon 27 oil (Sigma-Aldrich), covered with a coverslip and imaged. Live imaging of embryos was performed on a CSU10b Yokogawa spinning-disk confocal from Zeiss and Solamere Technologies Group with a 63×/1.4 NA objective. For slow movies, images were acquired in 30 s intervals with 27-30 *z*-slices at a 0.5 µm interval. Fast movies involved a single *z*-slice and 0.5-1 s intervals. For drug injection, after dechorionation as above, embryos were dehydrated for 15 min, covered with Halocarbon 700 oil and then injected with either colchicine (Sigma-Aldrich, C3915, 1 mg/ml in H_2_O) or dextran Alexa568 (Thermo Fisher Scientific, D22912). Embryos were imaged immediately after the injection.

### Embryo fixation, immunostaining and imaging

Embryos were collected on apple juice agarose plates and then dechorionated for 2 min before fixation. The embryos were fixed for 1 h 10 min at the interface of heptane and 4% formaldehyde in 0.1 M sodium phosphate buffer (pH 7.4). Then the embryos were manually devitellinized and stained with mouse anti-GFP (1:100; Molecular Probes, A-11120), mouse anti-Lamin (1:100; DSHB, ADL-195) or rabbit anti-Klp98A (1:100; Derivery et al., 2015). Secondary antibodies conjugated with Alexa 488 or Alexa 568 (Molecular Probes, A11001 or A11034) were used a 1:500 dilution. Embryos were mounted in Prolong Gold with DAPI (Life Technologies) to stain the nuclei for staging purposes. Immunostained embryos were imaged using an Olympus Fluoview FV1000 confocal laser scanning microscope with a 60×/1.42NA objective.

### Image editing and figure preparation

Spinning-disk and laser-scanning images were edited with ImageJ or Photoshop, and images were leveled identically between samples. All the graphs are generated in Graphpad Prism. Figures were prepared and labeled in Adobe Illustrator.

### Membrane furrow measurements

Embryos containing membrane (Resille:GFP or Spider:GFP) and histone (his2A:RFP) markers were live imaged. The point where apical membranes begin to meet and have a common width was determined to be the most apical *z*-layer. Progressive furrow ingression was measured by tracking the most basal point of a 4-5 ‘cell’ region over time. The morphology of the marked DNA was used to determine both stage and cell cycle status, where interphase was marked by the formation of new nuclei until the chromatin began to condense, prophase was defined as the period between chromosomal condensation and nuclear membrane disassembly, metaphase started after nuclear disassembly and ended at the beginnings of chromosomal segregation, and anaphase/telophase was the period between chromosomal segregation to the formation of new daughter nuclei (Xie and Blankenship, 2018).

### Nuclear fallout measurements

The amount of nuclear fallout in shRNA embryos was determined by counting the total amount of nuclei (marked with Histone:RFP) in the collected frame during one cycle and calculating the predicted amount for the next cycle by multiplying that value by two. The actual amount was measured for that cycle and then the percentage of remaining nuclei was calculated by dividing the actual value by the expected value.

### Colocalization quantification

Colocalization between Rab39 and Klp98A was performed on fixed images and colocalization between Rab39 and Rab35 and Rab35 and dextran was performed on live imaging. Rab39 or Rab35 puncta≥2 pixels in area were selected. The selected puncta were then overlaid with the opposing channel. When the overlapping region was equal to or larger than 2×2 pixels, the relationship between two proteins was determined as ‘colocalized’. Percent colocalization was calculated by dividing the number of colocalized puncta by the total number of puncta.

### Compartment velocity, intensity, and size measurements

Golgi and Rab39 velocity measurements were performed on time-lapse images. Rab39 or Golgi puncta equal to or larger than 2 pixels (Solamere spinning disk; pixel size=0.164 µm/pixel) were selected. The movement of the selected puncta was then tracked over time. Absolute velocity was defined by the distance between the initial position and the final position of a group of selected puncta after 1 min. When a given puncta moved more than 5 pixels (0.82 µm) in 10 s in a 1 min observation window, the movement was determined as rapid movement or ‘active movement’. The peak velocity was found by performing a division of the active movement length by the active movement duration. The percentage of events refers to the number of puncta moving actively relative to the total number of puncta. Compartment area and mean fluorescence intensity were determined using the free hand selection tool in ImageJ. Intensity values were obtained from images pre-edit and measured in ImageJ.

### Statistics and repeatability

Statistical significance was tested for using a paired, two-tailed Student's *t*-test unless otherwise stated. Not significant (ns): *P*>0.05; *P<0.05; **P<0.005; ***P<0.0005; ****P<0.00005. Error bars indicate standard error (s.e.m.).

## Supplementary Material

10.1242/develop.201547_sup1 Supplementary information
