# Supplementary material for: A Rab39-Klp98A-Rab35 endocytic recycling pathway is essential for rapid Golgi-dependent furrow ingression
Source: Development. 2023 Aug 17;150(16):dev201547. doi: 10.1242/dev.201547 (PMC10445802; doi:10.1242/dev.201547)
Supplement: Supplementary information [file develop-150-201547-s1.pdf]

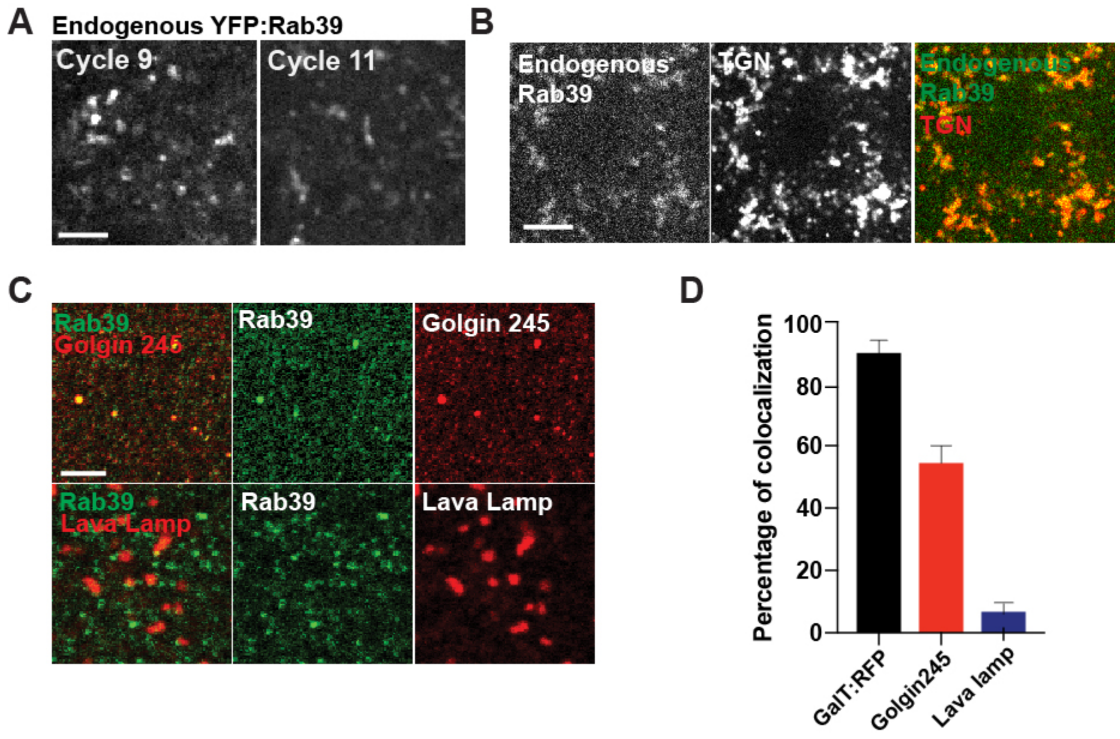

Fig. S1. Rab39 depletes over the syncytial stage and is colocalized with the trans Golgi (A) Still frames from live imaging of endogenous YFP-tagged Rab39 pre- and post-initiation of furrow formation (left, cycle 9 and, right, cycle 11) showing depletion of Rab39 size over time and similar dynamics to the UAS-YFP:Rab39 transgene (Fig. 1C). Scale bar = 5 µm. (B) Still frames from live imaging of endogenously expressed YFP:Rab39 and Galactoyltransferase:RFP, a marker of the trans Golgi network and merge. (C) Fixed images of YFP-tagged Rab39 embryos stained with anti-GFP and anti-Golgin 245 (top) or anti-GFP and Lava Lamp (bottom). Scale bar = 2.5µm. (D) Percentage colocalization of endogenous YFP:Rab39 with Galactosyltransferase:RFP, anti-Golgin245 (trans-Golgi marker co-stained with anti-GFP to detect YFP-Rab39), and anti-Lava Lamp (cis-Golgi marker co-stained with anti-GFP to detect YFP-Rab39) images shown in B and C in cycle 11 embryos. n=50 measurements for each indicated graph.

Development • Supplementary information

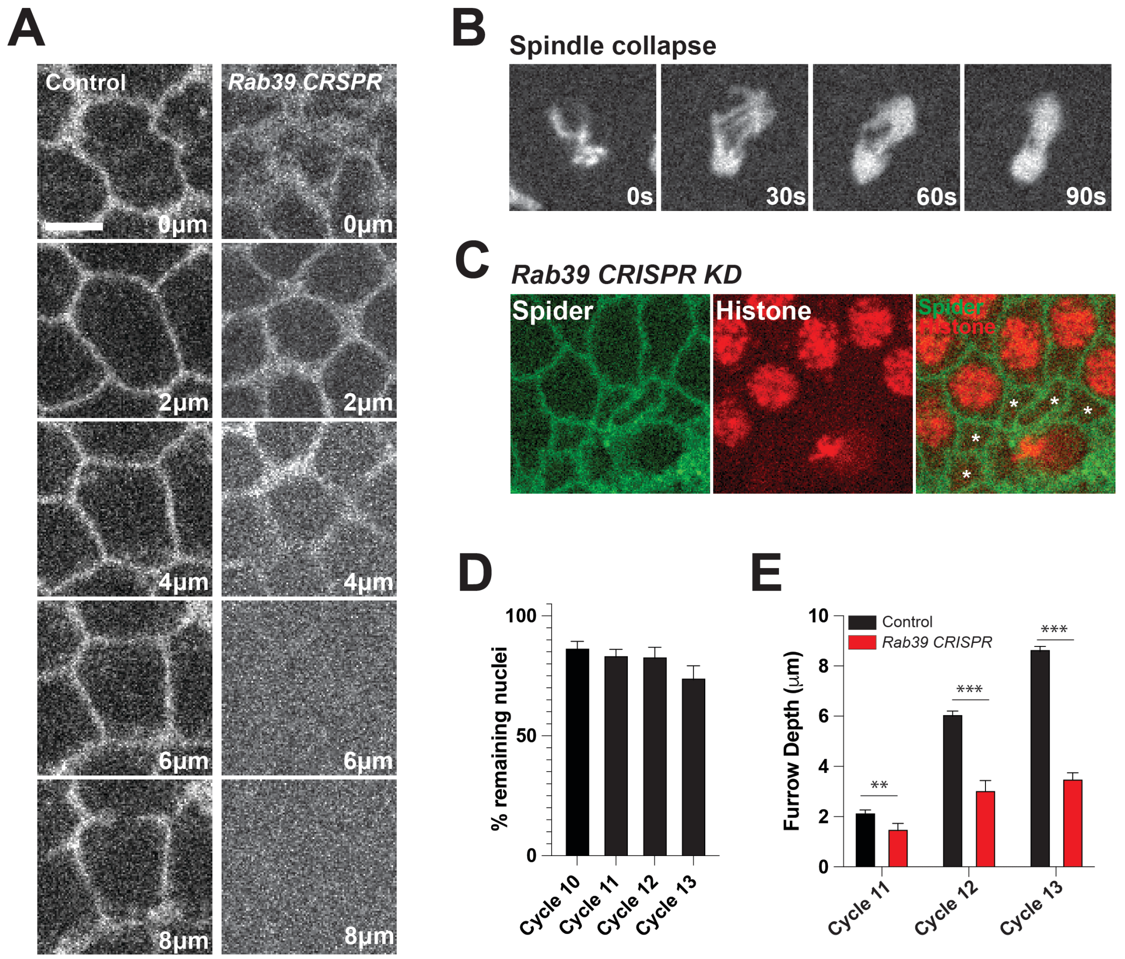

Fig. S2. CRISPR gRNA Rab39 expression results in similar phenotypes to *Rab39* shRNA depletion. (A) Still frames from live imaging of control and *Rab39 CRISPR gRNA* embryos of plasma membrane marker in cell cycle 13 at increasing depths. **(B)** Still frames from live imaging of dividing nuclei (His2av) in control and *Rab39 CRISPR gRNA* embryos in cell cycle 12. **(C)** Still images from live imaging of *Rab39 CRISPR gRNA* embryos in cell cycle 13 showing both plasma membrane (Spider) and Histone markers. Asterisks mark formation of 'mini-cells' as a result of nuclear fallout. **(D)** Quantitation of nuclear fallout by percent of remaining nuclei in each cell cycle. **(E)** Quantitation of maximum furrow depth in control and *Rab39 CRISPR gRNA* embryos in each cell cycle. Scale bar = 5 µm. Statistical significance has been calculated using Mann-Whitney U-test.

Development • Supplementary information

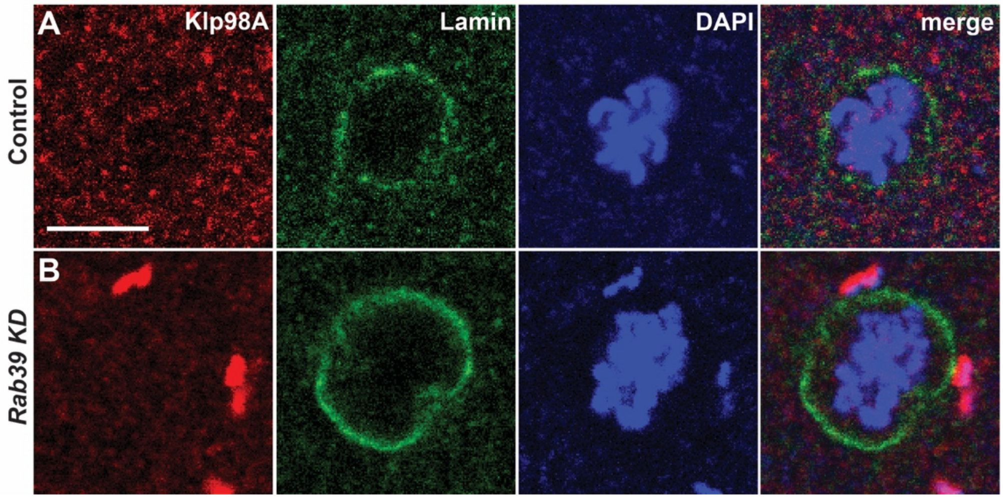

Fig. S3. Rab39 disruption results in a loss of Klp98A puncta and aberrant localization to fragmented chromosomes. (A,B) Fixed embryos in control (top) and Rab39 shRNA (bottom) backgrounds showing loss of punctate Klp98A signal in the early embryo. Note also that anti-Klp98A is found at cytoplasmic chromosomal fragments after *Rab39* disruption. Data from cycle 12 embryos. Scale bar = 5 µm.

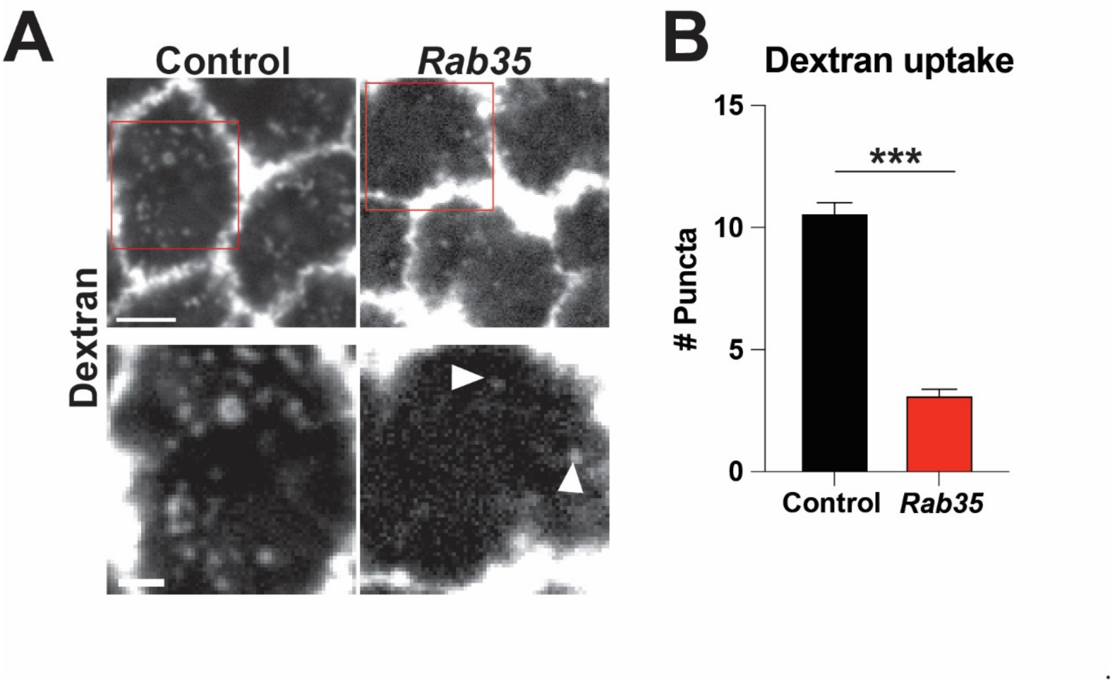

Fig. S4. *Rab35* depletion dramatically decreases the amount of internalized dextran. (A) Still images from live imaging of control and *Rab35* KD embryos injected with dextran into extracellular space. Bottom panel shows area of approximately one cell at cycle 11. **(B)** Quantification of number of dextran labeled puncta per 10µm x 10µm area in control and *Rab35 KD* embryos. Scale bars = 5 µm in top panel, 2 µm in bottom panel. Statistical significance has been calculated using Mann-Whitney U-test.

Development • Supplementary information

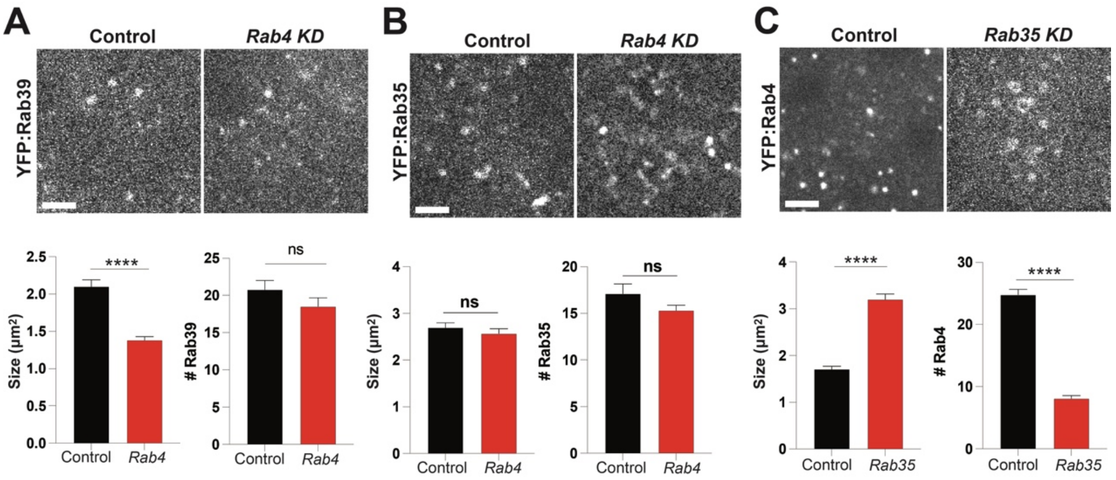

Fig. S5. Rab4 and Rab35 endosomes deliver membrane to Rab39 for recycling to Golgi. (A) Still images from live imaging of endogenous YFP:Rab39 in control and *Rab4* shRNA background (top). Average size in µm^2 is quantified (bottom, left) and number of Rab39 compartments (bottom, right) (number is compartments/100 µm^2). (B) Still images from live imaging of Rab35:YFP in control and *Rab4* shRNA background (top). Average size (bottom, left) and average number of Rab4 compartments (bottom, right) is quantified. (number is compartments/100 µm^2). (C) Still images from live imaging of Rab4:YFP in control and *Rab35* shRNA background. Average size in µm^2 (bottom, left) and average number of Rab4 compartments (bottom, right) (number is compartments/100 µm^2). Data from cycle 12 embryos. n=50. Scale bars = 5 µm. Statistical significance has been calculated using Mann-Whitney U-test.

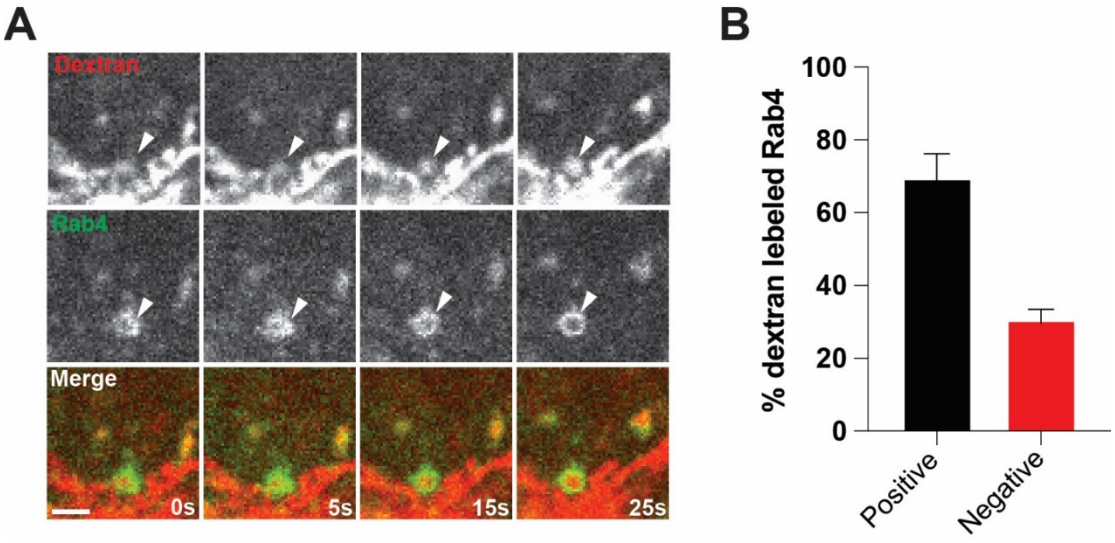

Fig. S6. Rab4 compartments contain extracellularly-derived dextran. (A) Time lapse images images from live imaging of YFP:Rab4 injected with dextran into the extracellular space. **(B)** Percentage of YFP:Rab4 puncta labeled with and without dextran. Images from cycle 11 embryo. Scale bar = 2 µm.

Development • Supplementary information

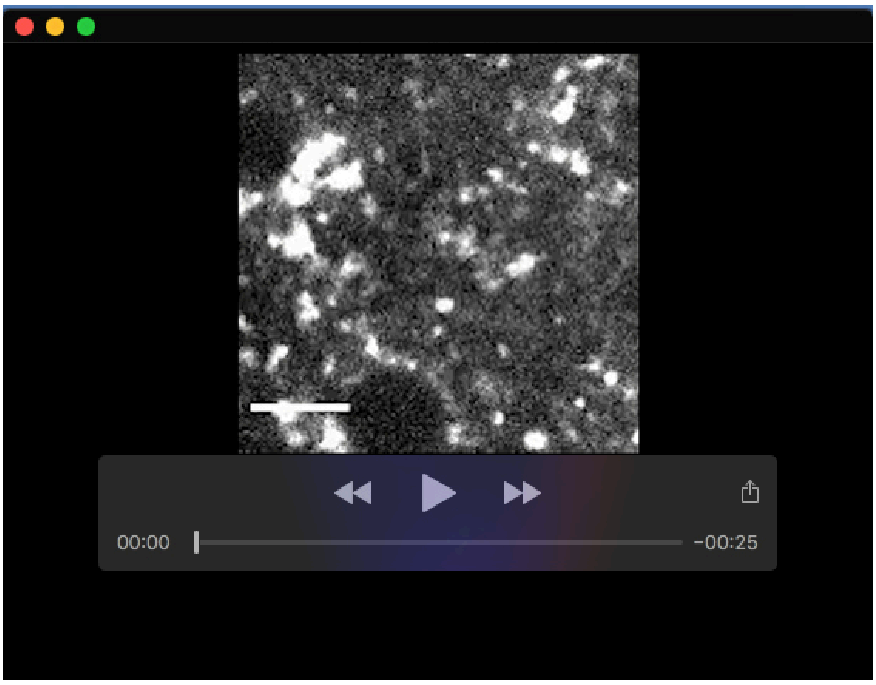

Movie 1. UAS-YFP:Rab39 at cycle 10 demonstrating rapid tubular and compartmental movements. Temporal resolution of 1 sec real time/frame acquisitions, and movie played at 12 frames/sec. Scale bar = 5 µm.

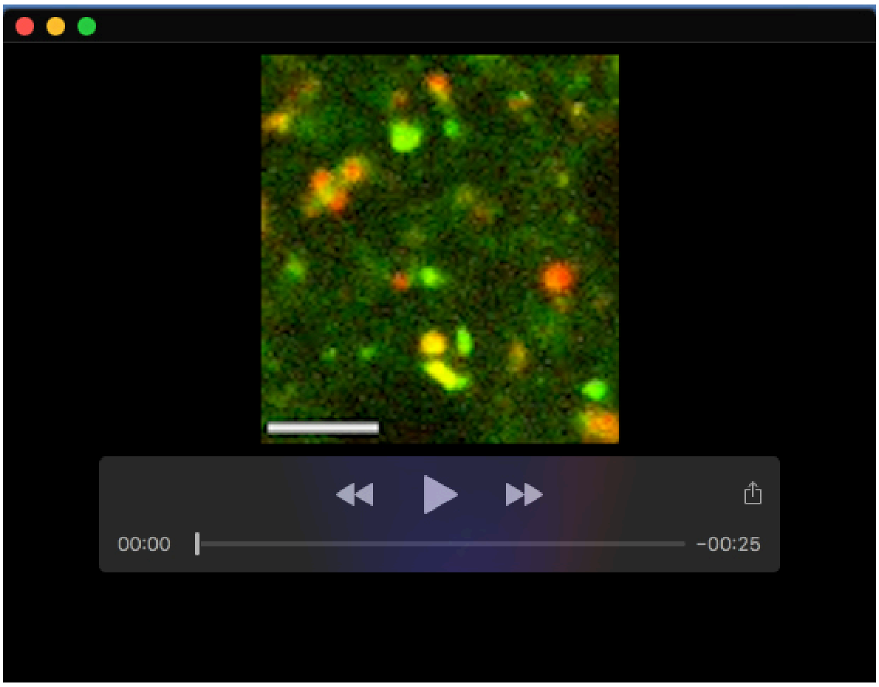

Movie 2. GalT:RFP and UAS-YFP:Rab39 at cycle 10. Temporal resolution of 1 sec real time/frame acquisitions, and movie played at 4 frames/sec. Scale bar = 5 µm.

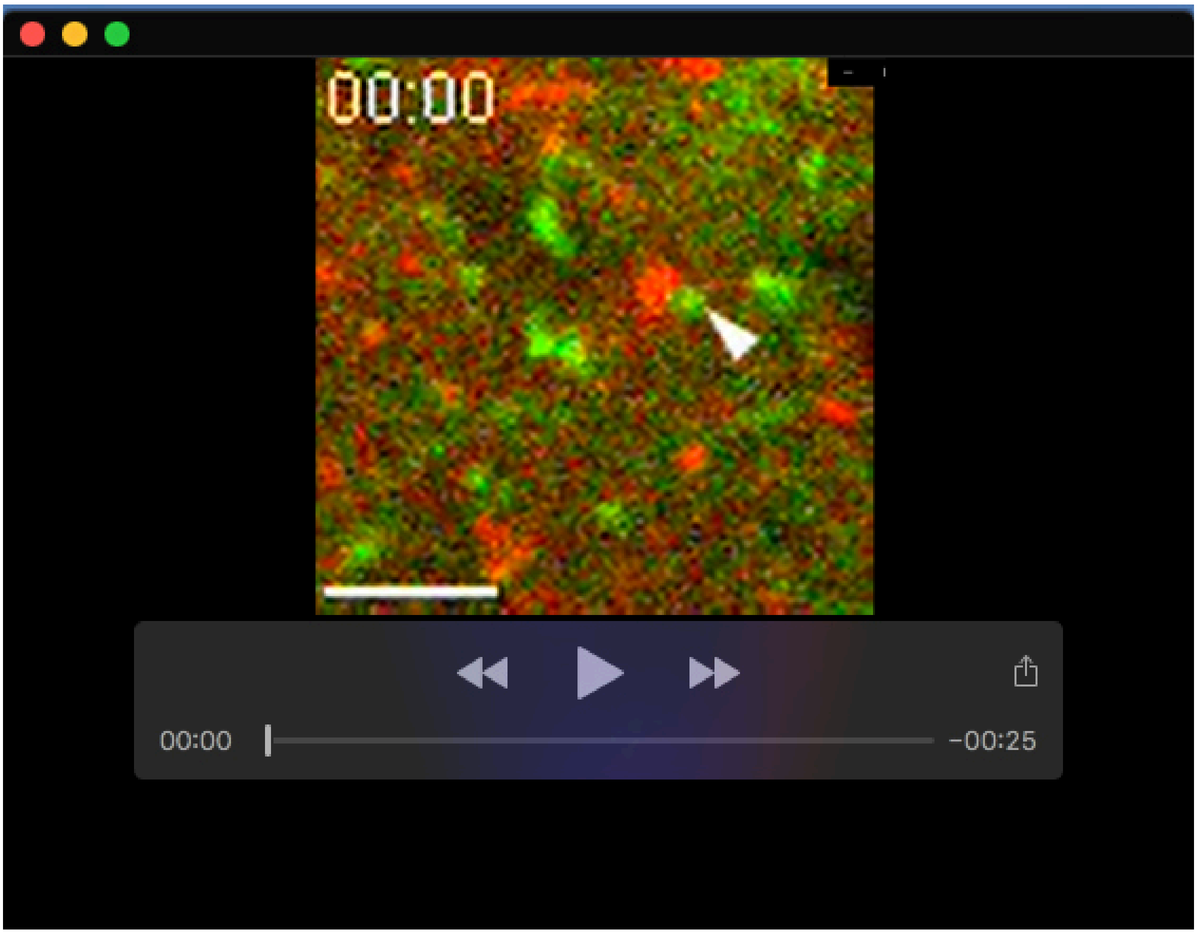

Movie 3. mCherry:Rab39 and YFP:Rab39 imaged at cycle 11. Temporal resolution of 1 sec real time/frame acquisitions, and movie played at 12 frames/sec. Scale bar = 5 µm.
